# Determinants of utilisation of antenatal care and skilled birth attendant at delivery in South West Shoa Zone, Ethiopia: a cross sectional study

**DOI:** 10.1186/s12978-015-0067-y

**Published:** 2015-08-25

**Authors:** Calistus Wilunda, Gianluca Quaglio, Giovanni Putoto, Risa Takahashi, Federico Calia, Desalegn Abebe, Fabio Manenti, Donata Dalla Riva, Ana Pilar Betrán, Andrea Atzori

**Affiliations:** Projects Department, Doctors with Africa CUAMM, Via San Francesco 126, Padua, Italy; Department of Pharmacoepidemiology, Graduate School of Medicine and Public Health, Kyoto University, Yoshida Konoecho, Sakyoku, Kyoto, 606-8501 Kyoto Japan; Department of Innovation, Research and Planning, Azienda ULSS 9, Treviso, Italy; Department of Nursing Science, Naragakuen University, 3-15-1, Nakatomigaoka, Nara-ishi, Nara Japan; Doctors with Africa CUAMM, P.O Box 12777, Addis Ababa, Ethiopia; St. Luke Hospital, P.O. Box 250, Wolisso, Ethiopia; Department of Reproductive Health and Research, World Health Organization, 1211 Geneva 27, Switzerland

## Abstract

**Background:**

Ethiopia has high maternal mortality ratio and poor access to maternal health services. Attendance of at least four antenatal care (ANC) visits and delivery by a skilled birth attendant (SBA) are important in preventing maternal deaths. Understanding the reasons behind the poor use of these services is important in designing strategies to address the problem. This study aimed to determine the coverage of at least four ANC visits and delivery by a SBA and to identify determinants of utilisation of these services in three districts in South West Shoa Zone, Ethiopia.

**Methods:**

A cross-sectional survey of 500 women aged 15–49 years with a delivery in two years prior to the survey was conducted in Wolisso, Wonchi and Goro districts in February 2013. Data were collected using an interviewer administered questionnaire. Logistic regression models were used to explore determinants of ANC attendance and SBA at delivery.

**Results:**

Coverage of at least four ANC visits and SBA at delivery were 45.5 and 28.6 %, respectively. Most institutional deliveries (69 %) occurred at the single hospital that serves the study districts. Attendance of at least four ANC visits was positively associated with wealth status, knowledge of the recommended number of ANC visits, and attitude towards maternal health care, but was negatively associated with woman’s age. SBA at delivery was negatively associated with parity and time to the health facility, but was positively associated with urban residence, wealth, knowledge of the recommended number of ANC visits, perceived good quality of maternal health services, experience of a pregnancy/delivery related problem, involvement of the partner/family in decision making on delivery place, and birth preparedness.

**Conclusions:**

Raising awareness about the minimum recommended number of ANC visits, tackling geographical inaccessibility, improving the quality of care, encouraging pregnant women to have a birth and complication readiness plan and community mobilisation targeting women, husbands, and families for their involvement in maternal health care have the potential to increase use of maternal health services in this setting. Furthermore, supporting health centres to increase uptake of institutional delivery services may rapidly increase coverage of delivery by SBA and reduce inequity.

**Electronic supplementary material:**

The online version of this article (doi:10.1186/s12978-015-0067-y) contains supplementary material, which is available to authorized users.

## Background

The fifth millennium development goal target is to reduce maternal mortality ratio (MMR) by 75 % between 1990 and 2015. Globally, it is estimated 289,000 maternal deaths occurred in 2013; a decline of 45 % from 1990 [1]. Sub-Saharan Africa accounted for 62 % of the global burden of maternal deaths. This region had the highest MMR at 500 maternal deaths per 100 000 live births compared to the global average of 210 maternal deaths per 100,000 live births [1]. Ethiopia has one of the highest MMRs worldwide: 676 per 100,000 live births [2]; positioning the country among the 10 countries that contribute to 58 % of all maternal deaths worldwide [1]. Ethiopia is also among the 10 countries with the highest numbers of intrapartum-related neonatal deaths and intrapartum stillbirths [3]. The country has a neonatal mortality rate 37 per 1000 live births, with little reduction since the year 2000 [2], and is one of the five countries that contribute to half of Africa’s newborn deaths [4].

Monitoring maternal mortality is expensive and technically demanding. Coverage indicators are therefore often used as good proxies to monitor mortality reduction. Increases in coverage signify that policies and delivery strategies are successfully reaching target populations [5]. The high maternal mortality in Ethiopia is reflective of the low coverage of quality maternal health services. Antenatal care (ANC) and skilled attendance at delivery are critical factors in preventing maternal deaths [6, 7]. Nevertheless, although, 42.6 % of women in Ethiopia attend at least one antenatal care visit, only 19.1 % complete the recommended minimum four visits. Moreover, only 10 % of deliveries are attended to by a skilled birth attendant (SBA) and the proportion of births by caesarean section is 1.5 % [2]; which is among the lowest rates worldwide and unlikely to cover the needs [8, 9]. Health system resources are scarce in Ethiopia: total health expenditure per capita per year is around 16 US dollars [10]. Even though the Ethiopian Government has in the past launched various Health Sector Development Programmes to address the health challenges in the county, such programmes have suffered from implementation problems [11].

Studies in Ethiopia have found that being in a rural area, having no education, being in lower wealth groups, being older, having a higher parity, staying far away from the health facility, not attending ANC, and not having knowledge about pregnancy related services are some of the factors associated with low utilisation of SBAs [12–18]. However, the role of some factors such as decision making regarding place of delivery, attitude towards maternal health care and birth preparedness have rarely been investigated. Additionally, although a number of studies have assessed determinants of receiving ANC in general in Ethiopia [15, 18–20]; there is lack of studies from the country that have explored determinants of attendance of at least four ANC visits.

This paper presents the results of a survey that was conducted in the context of a three year maternal and neonatal health project funded by the Italian International Cooperation in Wolisso, Goro and Wonchi districts (so-called *woredas* in Oromo language), and implemented by the non-governmental organisation Doctors with Africa CAUMM. The organisation has been supporting health services management and delivery in Ethiopia since 1984, and has adopted the continuum of care approach as its main health service delivery strategy [21]. The objectives of this survey were to determine the coverage of at least four ANC visits and delivery by a SBA, and to explore determinants of utilisation of these services in the three districts in Ethiopia.

## Methods

### Study design and setting

This is a cross sectional survey of women of reproductive age (15–49 years) who had delivered in the two years prior to the survey. The study was conducted in Wolisso, Goro and Wonchi districts of South West Shoa Zone, Oromiya region in central Ethiopia. The three districts have a combined population of about 372,478 inhabitants and are served by one hospital which also acts as a zonal referral hospital, 16 health centres (HCs), and 89 health posts. In Ethiopia, maternity services are usually provided at HCs as well as at hospitals by a health professional.

### Sample size and sampling

The sample size was estimated assuming institutional delivery coverage of 20 % based on the routine health data for the three districts, an absolute precision of 0.05, and a Z score value of 1.96 for 95 % confidence interval (CI). The sample size was further adjusted for a design effect of 2 yielding a minimum required sample size of 492. Multistage sampling using a modified Expanded Program for Immunisation’s random walk method [22] was used to select study subjects. The first stage involved selection of villages and the second stage involved selection of interviewees. First, the calculated sample size was allocated in proportion to the population in each district. Within each district, villages were randomly selected by probability proportionate to size. A total of twenty five villages were selected and 20 women were interviewed from each village. In each selected village, the centre was identified and while there, a pen was spun to identify the random walking direction. One team of data collectors began walking from the centre and visited consecutive households in the direction of the pen and another team went to the end of the village and visited consecutive houses towards the centre. One eligible woman was interviewed per household.

### Data collection and tools

Data were collected in February 2013 by trained data collectors utilising interviewer administered questionnaires that were adapted using the UNICEF’s Multiple Cluster Indicator Survey questionnaires and JHPIEGO’s tools for monitoring birth preparedness and complication readiness [23]. The questionnaires were pretested and translated into Oromo language. Two questionnaires were used: a household questionnaire which collected data on household characteristics, asset ownership and access to water and sanitation facilities; and a women’s questionnaire which collected data on characteristics of women and various aspects of maternal health care. Data were then entered in duplicate into EpiData version 3.1 and validated.

### Measures

The outcome variables were: (1) attendance of at least four ANC visits provided by a health professional or a health extension worker, and (2) delivery care by a SBA i.e. a doctor, nurse, midwife, or a health officer. Wealth index, which is a composite measure of a household’s cumulative living standard, was derived from factor analysis of household assets, housing material, and access to water and sanitation services [24, 25]. Attitude score was designed to assess attitude on three aspects of maternal health: birth preparedness, male involvement, and barriers to institutional delivery and it was derived from factor analysis of eight Likert-scale statements [23]. Barriers to institutional delivery focused on three aspects: costs, difficulties in getting to the health facility and handling of women by health facility staff. Male involvement focused on perception towards the husband accompanying his wife to the health facility for ANC and delivery, and the role of men in childbirth [23]. The scores were ranked into tertiles. Being well prepared for the birth of the baby was defined as having done any two of the following during pregnancy: identification of transport, saving money, identification of a blood donor, deciding on the facility where the baby will be born, and identification of a SBA [23].

### Data analysis

Data were analysed in Stata version 12 using survey commands to account for the complex sampling scheme by specifying the stratifying, clustering and weighting variables. Specifying the cluster and strata using the *svyset* command followed by use of the *svy* prefix with estimation commands in Stata adjusts for standard errors and produces confidence intervals and *p*-values that are unbiased by the survey design [26]. Weighting was performed using the inverse of the probability of selection in each residence (urban/rural), to correct for a slight oversampling in urban areas. Characteristics of participants were summarised using percentages. Because the sample size had been calculated to estimate prevalence as opposed to two sample comparisons (i.e. attendance of four ANC visits and delivery by a SBA; both dichotomous), post-hoc calculations of the power of the study to detect significant differences between comparison groups at 5 % level were performed. The results showed that the power was above 83 % for almost all the key variables (see additional file 1). Logistic regression models were used to obtain unadjusted and adjusted odds ratios with 95 % CIs for the associations between various factors and each of the outcome variables. Variables with *p* < 0.1 in unadjusted analysis were included in multivariate analysis which was performed using the forwards fitting approach [27]. The significance of each variable in the model was assessed using adjusted Wald test to obtain an F statistic and its associated *p* value. All *p* < 0.05 (2-sided) values were considered statistically significant.

### Ethical considerations

The study protocol and tools were approved by the Oromiya Regional Institutional Review Board and by the district health management teams of the study districts. Due to low literacy levels in the study setting, participants provided verbal informed consent after they had been introduced to the purpose of the study and informed about their right to interrupt the interview at any time or decline to be interviewed without any future prejudice.

## Results

### Characteristics of participants

Five hundred women participated in the survey and only three women approached for an interview (0.6 %) declined to participate. Among the participants, 45.5 % (95 % CI 38.0–53.0 %) attended at least four ANC visits (hereafter referred to as ANC) and 28.6 % (95 % CI 17.6–39.6 %) delivered with a SBA. Most women (54.4 %) were assisted at delivery by traditional birth attendants (TBAs) but a significant proportion (15.1 %) were assisted by relatives/friends with the remainder being assisted by health extension workers or no one. Among women who delivered in health facilities, 69.4 % delivered at the hospital, 27.1 % in HCs and the rest in other facilities. Table 1 presents a summary of participants’ characteristics. Most of the participants were from Wolisso District (55.6 %); rural dwellers (86.3 %); of Oromo ethnicity (86.6 %); uneducated (52.3 %); orthodox Christians (51.8 %); with a partner with at least primary education (77 %); and married (96.4 %). Less than a half (48.8 %) knew the minimum recommended number of ANC visits and 23.3 % could mention at least three danger signs of pregnancy. About 42.4 % stayed more than one hour from a health facility providing delivery service. Only 14.8 % perceived the quality of maternal health services at their nearest facility to be excellent; and 13.4 % were well prepared for delivery.

**Table 1:** Percent distribution of women by antenatal care attendance and delivery by skilled provider

Characteristics	Attended four ANC visits		Delivered by skilled provider		Total (*n* = 500)
	No (*n* = 263)	Yes (*n* = 237)	No (*n* = 335)	Yes (*n* = 165)	
District					
Wolisso	55.2	56.2	52.5	63.5	55.6
Goro	15.0	16.5	15.2	16.7	15.7
Wonchi	29.9	27.3	32.3	19.7	28.7
Urban residence	7.1	21.9	2.2	42.7	13.8
Non Oromo ethnicity	10.3	17.1	10.1	21.5	13.4
Household size					
2–3	13.2	21.1	10.9	31.5	16.8
4–5	33.2	37.0	35.0	34.9	34.9
>5	53.6	41.9	54.1	33.6	48.3
Wealth index quintile					
Lowest	26.5	11.8	25.4	6.0	19.8
Second	19.4	21.1	24.6	9.0	20.2
Middle	23.7	15.5	24.3	9.0	19.9
Fourth	18.8	21.8	18.0	25.6	20.2
Highest	11.6	29.8	7.7	50.3	19.9
Age in years					
15–24	21.4	32.2	22.8	35.0	26.3
25–29	34.5	40.5	38.6	33.7	37.2
30–34	22.2	14.3	19.4	16.7	18.6
35–49	22.0	13.0	19.2	14.6	17.9
Parity					
1	15.5	24.8	12.5	37.8	19.8
2–3	28.0	30.9	29.1	30.0	29.3
4–5	32.1	30.0	36.2	18.5	31.1
>5	24.4	14.3	22.2	13.7	19.8
Woman’s education level					
None	59.4	43.8	60.3	32.3	52.3
Primary	33.9	41.2	34.8	43.4	137.2
Secondary/higher	6.7	15.0	4.9	24.3	10.5
Partner’s education level					
None/no partner	27.2	17.9	28.5	9.2	23.0
Primary	57.2	56.4	59.5	50.2	56.8
Secondary/higher	15.6	25.7	12.0	40.6	20.2
Marital status					
Married	95.9	97.0	95.7	198.3	96.4
Single	4.1	3.0	4.3	1.7	3.6
Religion					
Orthodox Christian	54.0	49.2	53.0	48.9	51.8
Protestant	22.7	26.3	23.0	27.7	24.4
Muslim	23.3	24.5	24.0	23.4	23.8
Knows the required number of ANC visits	34.9	65.4	41.8	66.3	48.8
Knows ≥3 pregnancy danger signs	19.4	27.9	19.3	33.3	23.3
Time to nearest health facility					
<30 min	25.3	44.2	22.1	63.4	33.9
30–59 min	24.2	23.1	26.6	16.5	23.7
≥60 min	50.5	32.7	51.3	20.1	42.4
Perceived quality of care at nearest facility					
Average/poor/don’t know	44.2	34.2	43.5	30.0	39.7
Good	44.8	46.4	46.3	43.6	45.5
Excellent	11.0	19.4	10.1	26.4	14.8
Maternal health attitude score tertiles					
Poor	47.4	23.9	41.2	25.5	36.7
Medium	27.0	32.6	30.6	27.1	29.6
Good	25.5	43.5	28.2	47.4	33.7
Attended at least 4 ANC visits	--	--	36.2	68.9	45.5
Had any pregnancy/delivery problem	--	--	17.4	24.5	19.4
Well prepared for the birth of the baby	--	--	6.9	29.4	13.4
Final decider on delivery place					
Woman alone	--	--	48.3	23.1	41.1
Partner/other family member alone	--	--	34.0	60.3	41.5
Woman and partner together			17.7	16.6	17.4

### Determinants of antenatal care use

Results of the analysis of determinants of ANC use are presented in Table 2. In unadjusted analysis, only district, ethnicity, marital status and religion were not statistically significantly associated with ANC attendance. After adjusting for confounding factors, wealth index quintile, age, knowledge of the required number of ANC visits and maternal health attitude score maintained statistically significant associations with attending ANC. Wealth index and knowledge of the required number of ANC visits had the strongest associations with ANC attendance. Women in the highest wealth quintile had a three and a half fold increase in the odds of attending ANC compared to those in the lowest wealth quintile (OR 3.53, 95 % CI 1.69–7.39), and those who knew the recommended number of ANC visits had almost a threefold increase in the odds of attending ANC compared to those who did not know (OR 2.75, 95 % CI 1.89–4.01). The odds of attending ANC reduced with increasing age. Women with a good attitude towards maternal health were about twice more likely to attend ANC compared to those with a poor attitude (OR 2.20, 95 % CI 1.22–3.98).

**Table 2:** Odds ratios for the association between various factors and attendance of four ANC visits

Characteristics	Unadjusted OR (95 % CI)	*P* value^i^	Adjusted OR^a^ (95 % CI)	*P* value^i^
District		0.820		
Wolisso	1		--	
Goro	1.08 (0.58–2.01)		--	
Wonchi	0.89 (0.47–1.69)		--	
Urban residence	3.69 (2.21–6.17)	<0.001	1.62 (0.84–3.14)	0.145
Non Oromo ethnicity	1.79 (0.98–3.26)	0.058	1.41 (0.78–2.54)	0.241
Household size		0.017		0.867
2–3	2.04 (1.29–3.25)		1.14 (0.66–1.94)	
4–5	1.43 (0.91–2.24)		1.16 (0.61–2.21)	
>5	1		1	
Wealth index quintile		0.001		0.015
Lowest	1		1	
Second	2.44 (1.45–4.11)		2.29 (1.27–4.15)	
Middle	1.46 (0.75–2.85)		1.10 (0.50–2.41)	
Fourth	2.59 (1.50–4.47)		1.96 (1.04–3.68)	
Highest	5.74 (2.90–11.36)		3.53 (1.69–7.39)	
Age in years		0.001		0.003
15–24	1		1	
25–29	0.78 (0.50–1.21)		0.81 (0.47–1.38)	
30–34	0.43 (0.27–0.69)		0.37 (0.21–0.66)	
35–49	0.39 (0.24–0.64)		0.42 (0.25–0.72)	
Parity		0.030		0.936
1	1		1	
2–3	0.69 (0.42–1.13)		0.98 (0.54–1.76)	
4–5	0.58 (0.36–0.96)		1.07 (0.49–2.31)	
>5	0.37 (0.20–0.67)		0.89 (0.352.31)	
Woman’s education level		0.003		0.313
None	1		1	
Primary	1.65 (1.07–2.56)		1.00 (0.56–1.76)	
Secondary/higher	3.01 (1.70–5.33)		0.74 (0.40–1.35)	
Partner’s education level		0.015		0.770
None/no partner	1		1	
Primary	1.49 (0.86–2.59)		0.95 (0.49–1.84)	
Secondary/higher	2.48 (1.33–4.62)		0.80 (0.34–1.86)	
Marital status		0.514		
Married	1		--	
Single	0.72 (0.26–1.99)		--	
Religion		0.566		
Orthodox Christian	1		--	
Protestant	1.27 (0.80–2.03)		--	
Muslim	1.16 (0.71–1.87)		--	
Knows number of required ANC visits	3.53 (2.40–5.18)	<0.001	2.75 (1.89–4.01)	<0.001
Knows ≥3 pregnancy danger signs	1.61 (1.04–2.49)	0.032	0.90 (0.60–1.35)	0.605
Time to nearest facility		0.013		0.367
<30 min	1		1	
30–59 min	0.55 (0.29–1.03)		0.63 (0.31–1.29)	
≥60 min	0.37 (0.20–0 .69)		0.64 (0.32–1.29)	
Perceived quality of care at nearest facility		0.035		0.220
Average/poor/don’t know	1		1	
Good	1.34 (0.81–2.21)		1.46 (0.93–2.31)	
Excellent	2.28 (1.21–4.31)		1.72 (0.85–3.48)	
Maternal health attitude score tertiles		<0.001		0.033
Poor	1		1	
Medium	2.39 (1.54–3.69)		1.88 (1.11–3.18)	
Good	3.37 (1.93–5.86)		2.20 (1.22–3.98)	

^i^Adjusted Wald test *P*-value for the overall significance of the variable in the model

^a^adjusted for residence, knowledge of number of ANC visits, woman’s age wealth index quintile and maternal health attitude score

### Determinants of skilled birth attendant at delivery

Table 3 presents the results of the analysis of determinants of delivery with a SBA. In unadjusted analysis only district, ethnicity, marital status, religion or having a pregnancy/delivery related problem were not significantly associated with delivery by a SBA. After controlling for other factors, compared to rural dwellers, urban dwellers were more likely to deliver assisted by a SBA (OR 7.31, 95 % CI 2.88–18.55). Wealth index was positively associated with delivery by a SBA with women in the highest wealth quintile having a 9-fold increase in the odds of delivery by a SBA compared to those in the lowest wealth quintile (OR 8.94, 95 % CI 2.45–32.61). The odds of delivery by a SBA decreased with increasing parity and time to the nearest health facility. Unmarried women were less likely to deliver assisted by a SBA. Knowledge of the required ANC visits (OR 2.65, 95 % CI 1.47–4.76) and experience of a pregnancy/delivery problem (OR 2. 94, 95 % CI 1.31–6.61) were positively associated with delivery by a SBA. Women with an excellent perception about the quality of maternal health care at the nearest health facility were more likely to deliver assisted by a SBA compared to those who perceived the quality to be poor/average (OR 6.45, 95 % CI 2.77–15.01). Women who decided together with their husbands on delivery place had a 4-fold increase in the odds of delivery by a SBA than those who decided unilaterally. Women who were well prepared for the birth of the baby were more likely to deliver assisted by a SBA compared with those that were not well prepared (OR 4.71, 95 % CI 1.68–13.18).

**Table 3:** Odds ratios for the association between various factors and delivery by a skilled provider

Characteristics	Unadjusted OR (95 % CI)	*P* value^i^	Adjusted OR^a^ (95 % CI)	*P* value^i^
District		0.554		
Wolisso	1		--	
Goro	0.91 (0.26–3.13)		--	
Wonchi	0.51 (0.14–1.81)		--	
Urban residence	32.80 (12.40–86.74)	<0.001	7.31 (2.88–18.55)	<0.001
Non Oromo ethnicity	2.42 (0.62–9.49)	0.195	--	
Household size		<0.001		0.939
2–3	4.63 (2.77–7.75)		0.79 (0.15–4.10)	
4–5	1.60 (1.04–2.48)		0.79 (0.15–4.10)	
>5	1		1	
Wealth index quintile		<0.001		0.004
Lowest	1		1	
Second	1.54 (0.68–3.53)		0.76 (0.36–1.61)	
Middle	1.55 (0.67–3.61)		0.69 (0.19–2.49)	
Fourth	6.00 (2.63–13.69)		3.47 (1.46–8.25)	
Highest	27.46 (8.48–88.89)		8.94 (2.45–32.61)	
Age in years		0.026		0.262
15–24	1		1	
25–29	0.57 (0.37–0.87)		0.60 (0.28–1.27)	
30–34	0.56 (0.37–0.86)		0.54 (0.30–0.99)	
35–49	0.50 (0.26–0.96)		0.61 (0.27–1.38)	
Parity		<0.001		0.003
1	1		1	
2–3	0.34 (0.20–0.58)		0.30 (0.10–0.90)	
4–5	0.17 (0.10–0.28)		0.17 (0.06–0.44)	
>5	0.20 (0.09–0.45)		0.21 (0.07–0.66)	
Woman’s education level		<0.001		0.241
None	1		1	
Primary	2.33 (1.47–3.70)		0.52 (0.24–1.11)	
Secondary/higher	9.27 (3.01–28.48)		0.66 (0.19–2.22)	
Partner’s education level		<0.001		0.468
None/no partner	1		1	
Primary	2.49 (1.51–4.46)		0.67 (0.36–1.26)	
Secondary/higher	10.37 (5.16–20.85)		0.63 (0.25–1.60)	
Marital status		0.079		0.034
Married	1		1	
Single	0.39 (0.13–1.13)		0.22 (0.06–0.87)	
Religion		0.804		
Orthodox Christian	1		--	
Protestant	1.30 (0.58–2.91)		--	
Muslim	1.05 (0.40–2.76)		--	
Knows required number of ANC visits	2.74 (1.85–4.05)	<0.001	2.65 (1.47–4.76)	0.002
Knows ≥3 pregnancy danger signs	2.08 (1.16–3.75)	0.016	0.67 (0.30–1.52)	0.322
Time to nearest facility with maternity		0.001		0.023
<30 min	1		1	
30–59 min	0.22 (0.10–0.45)		0.48 (0.23–0.96)	
≥60 min	0.14 (0.05–0.37)		0.35 (0.15–0.82)	
Perceived quality of care at nearest facility		0.002		<0.001
Average/poor/don’t know	1		1	
Good	1.36 (0.81–2.31)		1.73 (0.87–3.47)	
Excellent	3.77 (1.89–7.49)		6.45 (2.77–15.01)	
Maternal health attitude score tertiles		0.004		0.104
Poor	1		1	
Medium	1.43 (0.81–2.53)		0.50 (0.24–1.01)	
Good	2.72 (1.58–4.66)		0.61 (0.31–1.25)	
Attended at least 4 ANC visits	3.91 (2.57–5.94)	<0.001	1.41 (0.93–2.17)	0. 105
Had a pregnancy/delivery related problem	1.54 (0.92–2.60)	0.090	2.94 (1.31–6.61)	0.011
Final decision maker on delivery place		<0.001		0.002
Woman alone	1		1	
Partner/other family member alone	1.97 (1.02–3.79)		4.76 (2.16–10.47)	
Woman and partner together	3.71 (2.45–5.63)		3.92 (1.64–9.36)	
Well prepared for delivery of baby	5.59 (2.42–12.85)	<0.001	4.71 (1.68–13.18)	0.005

^i^Adjusted Wald test *P*-value for the overall significance of the variable in the model

^a^adjusted for residence, partners education level, decider on place of delivery, attended 4 ANC visits, parity, wealth quintile, perceived quality of care at nearest facility, birth preparedness, marital status, had pregnancy/delivery problem, knowledge of the required number of ANC visits and time to the nearest health facility. Due to collinearity between age and parity, the final model excluded age; thus age is adjusted for all these variables except parity

## Discussion

In this study, attendance of at least four ANC visits was positively associated with wealth status, knowledge of the recommended ANC visits, and attitude towards maternal health care, but negatively associated with woman’s age. Delivery by a SBA was negatively associated with parity and time to the nearest health facility but was positively associated with urban residence, wealth, knowledge of the required number of ANC visits, having a better perception towards the quality of maternal health services, experience of a pregnancy/delivery related problem, and birth preparedness. Additionally, involvement of the partner/family in decision making on delivery place increased the likelihood of SBA at delivery but being unmarried reduced this likelihood. The higher coverages of at least four ANC visits (45.5 %) and SBA at delivery (28.6 %) in this study compared to the corresponding national averages of (19.1 and 10 %, respectively) may be partly because of the external donor support that the study districts have been receiving in the past years.

The findings that poverty and higher age are associated with reduced ANC attendance are consistent with those of a systematic review of 28 studies on determinants of ANC use [28] and can be interpreted as if women with previous pregnancy and birth experience don’t feel the need to go again through ANC for a new pregnancy. The present study however did not find significant associations between ANC attendance and many other socio-demographic factors as has been reported in other studies [14, 19, 28]. These studies looked at attendance of any ANC visit and not four or more ANC visits. Nevertheless, the discrepancies suggest that determinants of service access may vary by geographic location even within a country, and highlight the need to understand key determinants of service access in a given context in order to tailor intervention strategies [29]. Distance is a major determinant in the decision to seek care [30]. Although the Ethiopian government has recently tried to improve geographical accessibility in the study districts by building new HCs and opening new roads, 42 % of participants were staying more the 1 h from the nearest health facility, and utilisation of SBA, but not ANC, was being influenced by distance. This is because a woman in labour has little time within which to go to the hospital compared to woman an antenatal woman, and a usual walking distance may be unsurmountable for a woman in labour. Additionally, ANC but not SBA can be provided through outreach; reducing the geographical barrier. Knowledge of the recommended number of ANC visits was associated with increased use of ANC but knowledge of at least three pregnancy danger signs did not have significant effect on ANC attendance. Although both questions were meant to measure knowledge, the later might have been less specific; explaining the discrepancy. Although knowledge of safe motherhood practices such as danger signs of pregnancy may promote safer pregnancies and deliveries, few women knew at least 3 pregnancy danger signs; consistent with results previously reported [31, 32].

In line with results from previous studies in the same setting [33, 34], this study confirms the existence of two major dimensions of inequity in utilisation of SBA in the study districts: rural/urban location and wealth [35]. Although persistent disparities exist between poorer and richer women regarding utilisation of maternal and child health services in low income countries, SBA at delivery is the most inequitable [36, 37]. In general, services delivered through fixed health facilities such SBA at birth tend to show greater disparities than those which can be delivered through outreach such as ANC [38]. As in other studies [39], this study found an inverse relationship between utilisation of SBA and parity and confirms the need to target women with higher parity in maternal health service provision. Although based on small numbers, unmarried women were less likely to utilise SBA compared to married women. This may be because it is culturally unacceptable for an unmarried woman to become pregnant in this setting [40], consequently, unmarried women may be shying away from utilising maternal health services. The present study did not find statistically significant association between use of delivery service and woman’s age, woman’s education and partner’s education as has been reported elsewhere [39]. Our findings suggest that education is not a strong determinant of utilisation of maternal health services in this context where more than half of the women are uneducated.

Our study concurs with findings from other studies that have found that women are more likely to use delivery services if they experience pregnancy related problems [13, 14, 39, 41]. Women with complications in pregnancy may be referred by a TBA, other person assisting when the complication arose or even self-refer to health facilities. Unfortunately such referrals do not always happen on time and mothers/neonates have lost lives due to various delays [30]. In many resource-poor settings, ANC visits constitute one of the few times when women seek care presenting a critical opportunity for informing them about the importance of skilled delivery care. However, after accounting for other factors, attending at least four ANC visits was not associated with increased SBA at delivery in our study as has been reported elsewhere [42].

In the present study, women whose final decision on where to deliver was made in consultation with the partner or by a family member were more likely to be assisted by a SBA at delivery. Similar findings have been reported in a study conducted in the same region [13], and highlight the importance of family support in improving maternal health services utilisation. Although women’s autonomy increases health service utilisation [43, 44], the role of family concern and support especially for SBA utilisation cannot be overlooked. On the other hand, studies have also shown the negative effect of too many people being involved in the decision making process leading to delays in seeking care [41], an effect that does not seem to be applicable to this area of Ethiopia.

Having a birth plan increases the likelihood of delivering in a health facility [45]. Theoretically, this is mediated through reduction in delays in obtaining care [30]. Our findings that women who were well prepared for the delivery of the baby were more likely to be assisted by a SBA at delivery support the concept of birth preparedness and complication readiness during pregnancy. Unfortunately, in line with the present study, a worldwide systematic review [41] and studies in Ethiopia [46, 47] have found that few women are well prepared for birth and pregnancy complications. This problem is aggravated by poor knowledge and practices related to birth preparedness [47].

Perceived low quality of care is a major barrier to utilisation of maternal health services and can result into the first delay: delaying the decision to seek care [30, 41]. The findings in the present study are in line with this well documented barrier and highlight the need to simultaneously address both the demand and supply side barriers to improve service utilisation [48].

This survey has limitations that prevent the full understanding of the barriers and facilitators for ANC attendance and delivery assisted by SBA in the study area. It is not only the perceived quality of care provided at health facilities that is important. With the ultimate objective of improving maternal and newborn health, the real quality of the care, whether antenatal care includes evidence-based interventions and to what extent equipment, tests, medicines and other supplies are available are paramount. Transportation and direct or indirect costs were also not evaluated in this survey. Inadequate staffing potentially leading to long waiting time in receiving care at health facilities was also not studied here.

## Conclusions

This is the first study to assess determinants of use of two important interventions for reducing maternal and newborn mortality and morbidity during pregnancy and childbirth in Goro, Wolisso and Wonchi districts. The findings highlight the importance of raising awareness about ANC and emphasising the minimum recommended number of ANC visits. All pregnant women should be encouraged to have a birth and complication readiness plan. This could be done routinely during ANC visits and also through community mobilisation with the involvement of health extension workers. At the moment, the study districts have numerous HCs whose utilisation is still very low; only 27 % of women delivered in 16 HCs as compared to 69 % who delivered in the one hospital. There is a high potential of improving coverage of delivery by SBA in the study districts if HCs are strengthened to respond to the maternal health needs in the population. Apart from increasing coverage, improving service delivery at HCs will contribute towards reduced inequity. A recent study has found huge gaps in the availability of emergency obstetric care services at HCs [33]; further justifying the need for more support at this level. The problem of long distance to the health facility could be addressed through innovative solutions such as use of transport vouchers [49] or other transportation schemes, and construction of health centres in underserved areas. The important role of the family found in this survey highlights the need not only to mobilise women but also their husbands, families and the entire community. Pregnant women need the encouragement and support of family members in order to access delivery care service. Given that most women deliver at home assisted by TBAs, there is need for collaboration between the formal health system and TBAs with the aim of encouraging TBAs to refer pregnant women to deliver in health facilities.

## Additional file

Additional file 1:
**Table: Post-hoc power calculations based on various variables for attendance of at least four antenatal care (ANC) visits and delivery by skilled birth attendant (SBA).** (DOCX 17 kb)

## Abbreviations

ANC: Antenatal care
CI: Confidence interval
CUAMM: *Collegio Universitario Aspiranti Medici Missionari*
HC: Health centre
JHPIEGO: Johns Hopkins Program for International Education in Gynaecology and Obstetrics
MMR: Maternal mortality ratio
OR: Odds ratio
SBA: Skilled birth attendant
TBA: Traditional birth attendant
UNICEF: United Nations Children’s Fund

## References

[1] WHO, UNICEF, UNFPA, World Bank, UN (2014). Trends in maternal mortality: 1990 to 2013 Estimates by WHO, UNICEF, UNFPA, The World Bank and the United Nations Population Division.

[2] Central Statistical Agency [Ethiopia], ICF International (2012). Ethiopia Demographic and Health Survey 2011.

[3] Lawn JE, Lee AC, Kinney M, Sibley L, Carlo WA, Paul VK (2009). Two million intrapartum-related stillbirths and neonatal deaths: Where, why, and what can be done?. Int J Gynaecol Obstet.

[4] The Partnership for Maternal Newborn and Child Health (2006). Opportunities for Africa’s Newborns Practical data, policy and programmatic support for newborn care in Africa.

[5] UNICEF (2005). Tracking progress in child survival: The 2005 report.

[6] Bhutta ZA, Das JK, Bahl R, Lawn JE, Salam RA, Paul VK (2014). Can available interventions end preventable deaths in mothers, newborn babies, and stillbirths, and at what cost?. Lancet.

[7] Ronsmans C, Graham WJ (2006). Maternal mortality: who, when, where, and why. Lancet.

[8] Gibbons L, Belizan JM, Lauer JA, Betran AP, Merialdi M, Althabe F (2012). Inequities in the use of cesarean section deliveries in the world. Am J Obstet Gynecol.

[9] Betran AP, Merialdi M, Lauer JA, Bing-Shun W, Thomas J, Van Look P (2007). Rates of caesarean section: analysis of global, regional and national estimates. Paediatr Perinat Epidemiol.

[10] Tesfay R, Accorsi S, Akalu T, Vella V, Mamo D (2010). Assessing the performance of the health sector: achievements and challenges in EFY 2002. FMOH Quarterly Health Bull.

[11] Federal Ministry of Health (2008). Ethiopia Health Sector Development Program III 2005/06–2010/11: Mid-Term Review.

[12] Abebe F, Berhane Y, Girma B (2012). Factors associated with home delivery in Bahirdar, Ethiopia: a case control study. BMC Res Notes.

[13] Fikre AA, Demissie M (2012). Prevalence of institutional delivery and associated factors in Dodota Woreda (district), Oromia regional state, Ethiopia. Reprod Health.

[14] Tsegay Y, Gebrehiwot T, Goicolea I, Edin K, Lemma H, Sebastian MS (2013). Determinants of antenatal and delivery care utilization in Tigray region, Ethiopia: a cross-sectional study. Int J Equity Health.

[15] Birmeta K, Dibaba Y, Woldeyohannes D (2013). Determinants of maternal health care utilization in Holeta town, central Ethiopia. BMC Health Serv Res.

[16] Mekonnen MG, Yalew KN, Umer JY, Melese M (2012). Determinants of delivery practices among Afar pastoralists of Ethiopia. Pan Afr Med J.

[17] Amano A, Gebeyehu A, Birhanu Z (2012). Institutional delivery service utilization in Munisa Woreda, South East Ethiopia: a community based cross-sectional study. BMC Pregnancy Childbirth.

[18] Tarekegn SM, Lieberman LS, Giedraitis V (2014). Determinants of maternal health service utilization in Ethiopia: analysis of the 2011 Ethiopian Demographic and Health Survey. BMC Pregnancy Childbirth.

[19] Regassa N (2011). Antenatal and postnatal care service utilization in southern Ethiopia: a population-based study. Afr Health Sci.

[20] Fekede B, G/Mariam A (2007). Antenatal care services utilization and factors associated in Jimma Town (south west Ethiopia). Ethiop Med J.

[21] Getahun H, Berhane Y (2000). Abortion among rural women in north Ethiopia. Int J Gynaecol Obstet.

[22] Bostoen K, Chalabi Z (2006). Optimization of household survey sampling without sample frames. Int J Epidemiol.

[23] JHPIEGO (2004). Monitoring birth preparedness and complication readiness: tools and indicators for maternal and newborn health.

[24] Rutstein S, Johnson K (2004). The DHS Wealth Index. DHS Comparative Reports No. 6.

[25] Filmer D, Pritchett LH (2001). Estimating wealth effects without expenditure data--or tears: an application to educational enrollments in states of India. Demography.

[26] StataCorp (2013). Stata survey reference manual release 13.

[27] Kirkwood B, Sterne J (2003). Essential Medical Statistics.

[28] Simkhada B, Teijlingen ER, Porter M, Simkhada P (2008). Factors affecting the utilization of antenatal care in developing countries: systematic review of the literature. J Adv Nurs.

[29] Hodgins S (2013). Achieving better maternal and newborn outcomes: coherent strategy and pragmatic, tailored implementation. Glob Health Sci Pract.

[30] Thaddeus S, Maine D (1994). Too far to walk: maternal mortality in context. Soc Sci Med.

[31] Pembe AB, Urassa DP, Carlstedt A, Lindmark G, Nystrom L, Darj E (2009). Rural Tanzanian women’s awareness of danger signs of obstetric complications. BMC Pregnancy Childbirth.

[32] Hailu M, Gebremariam A, Alemseged F (2010). Knowledge about obstetric danger signs among pregnant women in Aleta Wondo District, Sidama Zone, Southern Ethiopia. Ethiop J Health Sci.

[33] Wilunda C, Putoto G, Dalla Riva D, Manenti F, Atzori A, Calia F (2015). Assessing coverage, equity and quality gaps in maternal and neonatal care in sub-saharan Africa: an integrated approach. PLoS One.

[34] Wilunda C, Putoto G, Manenti F, Castiglioni M, Azzimonti G, Edessa W (2013). Measuring equity in utilization of emergency obstetric care at Wolisso Hospital in Oromiya, Ethiopia: a cross sectional study. Int J Equity Health.

[35] Hosseinpoor AR, Bergen N, Koller T, Prasad A, Schlotheuber A, Valentine N (2014). Equity-oriented monitoring in the context of universal health coverage. PLoS Med.

[36] Boerma JT, Bryce J, Kinfu Y, Axelson H, Victora CG (2008). Mind the gap: equity and trends in coverage of maternal, newborn, and child health services in 54 Countdown countries. Lancet.

[37] Barros AJ, Ronsmans C, Axelson H, Loaiza E, Bertoldi AD, Franca GV (2012). Equity in maternal, newborn, and child health interventions in Countdown to 2015: a retrospective review of survey data from 54 countries. Lancet.

[38] Bhutta AZ, Chopra M, Axelson H, Berman P, Boema T, Bryce J (2010). Countdown to 2015 decade report (2000–10): taking stock of maternal, newborn, and child survival. Lancet.

[39] Gabrysch S, Campbell OM (2009). Still too far to walk: literature review of the determinants of delivery service use. BMC Pregnancy Childbirth.

[40] Harborview Medical Center. Ethiopian cultural profile. Harborview Medical Center. 2008. https://ethnomed.org/culture/ethiopian/copy_of_ethiopian-cultural-profile. Accessed 26.07.2015.

[41] Bohren MA, Hunter EC, Munthe-Kaas HM, Souza JP, Vogel JP, Gulmezoglu AM (2014). Facilitators and barriers to facility-based delivery in low- and middle-income countries: a qualitative evidence synthesis. Reprod Health.

[42] Adjiwanou V, Legrand T (2013). Does antenatal care matter in the use of skilled birth attendance in rural Africa: a multi-country analysis. Soc Sci Med.

[43] Mistry R, Galal O, Lu M (2009). Women’s autonomy and pregnancy care in rural India: a contextual analysis. Soc Sci Med.

[44] Woldemicael G, Tenkorang EY (2010). Women’s autonomy and maternal health-seeking behavior in Ethiopia. Matern Child Health J.

[45] Magoma M, Requejo J, Campbell O, Cousens S, Merialdi M, Filippi V (2013). The effectiveness of birth plans in increasing use of skilled care at delivery and postnatal care in rural Tanzania: a cluster randomised trial. Trop Med Int Health.

[46] Hailu M, Gebremariam A, Alemseged F, Deribe K (2011). Birth preparedness and complication readiness among pregnant women in Southern Ethiopia. PLoS One.

[47] Hiluf M, Fantahun M (2008). Birth preparedness and complication readiness among women in Adigrat town, north Ethiopia. Ethiop J Health Dev.

[48] Amudhan S, Mani K, Rai SK, Pandav CS, Krishnan A (2013). Effectiveness of demand and supply side interventions in promoting institutional deliveries--a quasi-experimental trial from rural north India. Int J Epidemiol.

[49] Van de Poel E, Flores G, Ir P, O’Donnell O, Van Doorslaer E (2014). Can vouchers deliver? An evaluation of subsidies for maternal health care in Cambodia. Bull World Health Organ.

