# Supplementary material for: Determinants of utilisation of antenatal care and skilled birth attendant at delivery in South West Shoa Zone, Ethiopia: a cross sectional study
Source: Reprod Health. 2015 Aug 25;12:74. doi: 10.1186/s12978-015-0067-y (PMC4592558; doi:10.1186/s12978-015-0067-y)
Supplement: Additional file 1: — Table: Post-hoc power calculations based on various variables for attendance of at least four antenatal care (ANC) visits and delivery by skilled birth attendant (SBA). (DOCX 17 kb) [file 12978_2015_67_MOESM1_ESM.docx]

**Additional file**

**Table: Post-hoc power calculations based on various variables for attendance of at least four antenatal care (ANC) visits and delivery by skilled birth attendant (SBA)**

| **Variable** | **Categories** | **Power** | |
| --- | --- | --- | --- |
|  |  | **ANC** | **SBA** |
| Residence | Rural versus Urban | 99.9% | 100% |
| Wealth index | Lowest versus Highest | 100% | 100% |
| Age in years | 15-24 versus 35-49 | 90.8% | 60.6% |
| Parity | 1 versus >5 | 91.8% | 99.9% |
| Woman’s education level | None versus Secondary/higher | 96.2% | 100% |
| Partner’s education level | None versus Secondary/higher | 89.6% | 100% |
| Time to nearest HF | <30 min vs >60 min | 99.8% | 100% |
| Knows >3 pregnancy danger signs | No versus Yes | 59.5% | 89.6% |
| Knows the required number of ANC visits | No versus Yes | 100% | 99.8% |
| Attitude score tertile | Poor(lowest) versus Good (highest) | 99.9% | 98.4% |
| Perceived quality of care at nearest HC/Hospital | Average/poor versus Excellent | 83.2% | 99.5% |
| Attended at least 4 ANC visits | No versus Yes | -- | 100% |
| Had any pregnancy/delivery problem | No versus Yes | -- | 39.8% |
| Well prepared for the birth of the baby | No versus Yes | -- | 100% |
| Final decider on delivery place | Woman alone versus Woman and partner together | -- | 100% |
